# Refractory Severe Thrombocytopenia during Pregnancy: How to Manage

**DOI:** 10.1055/s-0038-1675186

**Published:** 2018-12

**Authors:** Joana Gomes de Amorim, Manuel Rocha Abecasis, Filipa Maria Nogueira Lança Rodrigues

**Affiliations:** 1 Department of Anaesthesiology, Centro Hospitalar Lisboa Norte, Lisboa, Portugal

**Keywords:** pregnant woman, thrombocytopenia, idiopathic thrombocytopenic purpura, thromboelastometry, hemorrhage, grávida, trombocitopenia, púrpura trombocitopénica idiopática, tromboelastometria, hemorragia

## Abstract

Thrombocytopenia is the most common hemostatic change in pregnancy, but severe thrombocytopenia is rare. One of the causes, immune thrombocytopenic purpura (ITP), is characterized by increased platelet destruction by immunoglobulin G (IgG) antibodies, presenting a high risk of hemorrhage for the patient, but also for the fetus, since antibodies may cross the placenta. We present the case of a 23-year-old pregnant woman with a history of Langerhans cell histiocytosis of the mandible submitted to surgery and chemotherapy when she was 10 years old, with diagnosis of ITP since then. At 28 weeks of gestation, she presented with petechiae, epistaxis, and gingival bleeding, with a platelet count of 3 × 10^9^/L and positive IgG antiplatelet antibodies test. At a multidisciplinary discussion, it was decided to delay a cesarean section, due to the absence of fetal distress and to the high risk of morbidity for the patient. Many therapies were attempted without success. The IgG produced a slight and transient increase in the platelet count. On the 36^th^ week of gestation, an elective cesarean section was performed. The perioperative period transfusions were guided by rotational thromboelastometry (ROTEM) monitoring. The procedure was performed under general anesthesia and videolaryngoscopy-assisted intubation. The patient was hemodynamically stable, without significant bleeding, and was transferred to the intensive care unit. The platelet count eventually decreased and a splenectomy was performed. Regional anesthesia may be contraindicated, and general anesthesia is associated with an increased risk of airway hemorrhage due to traumatic injury during the tracheal intubation and of hemorrhage associated with the surgical procedure. A multidisciplinary approach is essential in high-risk cases.

## Resumo

A trombocitopenia é a alteração da hemostase mais comum na gravidez. Contudo, a trombocitopenia grave é rara. Uma das suas causas, a púrpura trombocitopênica imunológica (PTI), é caracterizada pelo aumento da destruição plaquetária por anticorpos de imunoglobulina G (IgG), apresentando alto risco de hemorragia para a paciente e também para o feto, uma vez que os anticorpos podem atravessar a placenta. Apresentamos o caso de uma grávida de 23 anos com histórico de histiocitose de células de Langerhans da mandíbula submetida a cirurgia e quimioterapia aos 10 anos de idade, com diagnóstico de PTI desde então. Na 28^a^ semana de gestação, a paciente apresentou um quadro de petéquias, epistaxe e hemorragia gengival, com contagem plaquetária de 3 × 10^9^/L e teste de anticorpos antiplaquetários IgG positivo. Em uma discussão multidisciplinar, decidiu-se adiar a cesariana devido à ausência de sofrimento fetal e ao alto risco de morbidade para a paciente. Muitas terapêuticas foram tentadas sem sucesso. A IgG produziu apenas um ligeiro e transitório aumento na contagem plaquetária. Na 36ª semana de gestação, foi realizada uma cesariana eletiva. As transfusões no período perioperatório foram guiadas por tromboelastometria rotacional (ROTEM). O procedimento foi realizado sob anestesia geral e intubação assistida por videolaringoscopia. A paciente manteve-se hemodinamicamente estável, sem hemorragia significativa. Ela foi transferida para a unidade de terapia intensiva. A contagem plaquetária continuou a diminuir, e a paciente foi submetida a uma esplenectomia. Nestes casos, a anestesia regional pode ser contraindicada, e a anestesia geral está associada a um risco aumentado de hemorragia das via aéreas devido a lesão traumática durante a intubação traqueal e de hemorragia associada ao procedimento cirúrgico. Uma abordagem multidisciplinar é essencial em casos de alto risco.

## Introduction

Thrombocytopenia is the most common hemostatic change during pregnancy, occurring in ∼ 7 to 10% of the pregnant women. However, severe thrombocytopenia is rare, and occurs in < 0.1% of the pregnant women.^1^

Among the different etiologies is immune thrombocytopenic purpura (ITP), which is an autoimmune disease characterized by the existence of antiplatelet antibodies that bind to the platelet membrane and induce their destruction in the reticuloendothelial system. Immune thrombocytopenic purpura represents 3% of the cases of severe thromobocytopenia.^2^ ^3^

Although it does not affect the normal fetal development, this pathology presents a high risk of morbidity and mortality not only for the pregnant woman, given the high risk of maternal hemorrhage, especially in childbirth, but also for the fetus. Antiplatelet antibodies cross the placenta and may cause neonatal thrombocytopenia.^4^ ^5^

The objective of the present case report is to present our multidisciplinary approach, experience and decision-making process concerning a pregnant woman with ITP refractory to treatment, considering the best risk-benefit relationship, regarding the ideal timing for the performance of the cesarean section.

## Case Report

We present the case of a 23-year-old pregnant woman, G1P0, with a history of histiocytosis of Langerhans cells of the mandible when she was 10 years old, submitted to resection surgery and adjuvant chemotherapy (prednisone, vimblastine, and methotrexate). At the time of this episode, ITP was diagnosed. Since then, the patient did not have regular medical follow-ups or medication, was asymptomatic and had platelet counts between 30 and 70 × 10^9^/L.

At 28 weeks of gestation, the patient presented to the emergency department due to petechiae on the limbs, epistaxis, and gingival bleeding, and presented a platelet count of 3 × 10^9^/L. She was admitted to the internal medicine ward, where she was treated for 9 days. Therapy included prednisolone (1 mg/kg/day) and immunoglobulin G (IgG) (20 g/day) for 5 days, which resulted in an increase in the platelet count to 49 × 10^9^/L. The tests for antiplatelet antibodies for glycoproteins IIb/IIIa and Ia/IIa were positive. After the treatment, the patient was asymptomatic and was discharged.

Seven days after the discharge, the patient returned to the emergency department with overlapping symptoms and aggravation of thrombocytopenia (with a platelet count < 10 × 10^9^/L). A multidisciplinary discussion was held, involving obstetrics, hematology, anesthesiology, neonatology and immunohemotherapy teams, and an elective cesarean section was proposed at 28 weeks and 1 day of gestation. However, given the absence of fetal distress and the high perioperative morbidity and mortality risk for the patient (at the date with a platelet count of 3 × 10^9^/L), the procedure was delayed, and she was hospitalized for optimization of thrombocytopenia.

**Fig. 1:**
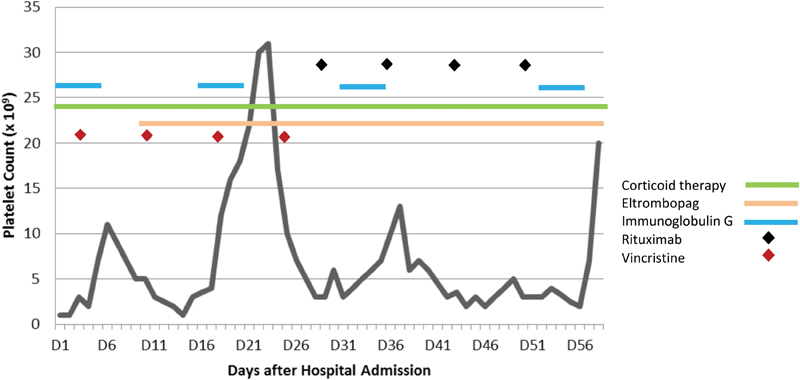
Variation of platelet count and relation with targeted therapy during hospital admission.

During hospitalization, the patient underwent 55 days of corticoid therapy, 46 days of Eltrombopag, 4 cycles of Vincristine, 4 cycles of Rituximab, and 5 cycles of IgG. Only the latter agent elicited a slight and transient response, resulting in a maximum platelet count of 3 × 10^9^/L, at 31 weeks of gestation (Fig. 1). Throughout this period, the patient remained clinically stable and deprived of any signs of hemorrhagic dyskinesia. The myelogram revealed increased megakaryocytes with no atypical cells.

**Fig. 2:**
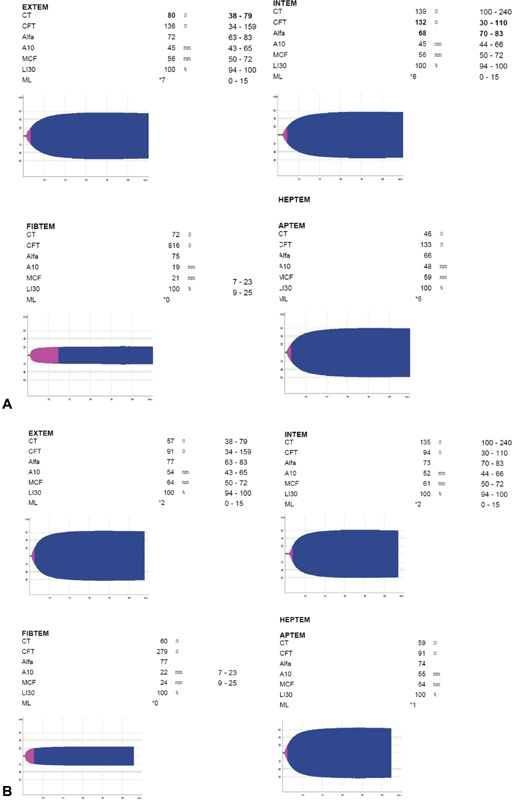
Rotational thromboelastometry result before (A) and after (B) cesarean section.

At the 36^th^ week of pregnancy, after the end of the 5^th^ cycle of IgG, an informed consent was obtained and an elective cesarean section was performed, with a platelet count of 20 × 10^9^/L. Rotational thromboelastometry (ROTEM) was performed in the immediate preoperative period (Fig. 2), which revealed a slight prolongation of the clot formation time (CFT) and a reduction of the α angle with maximum clot firmness (MCF) in the lower limit of normality in Intrinsically-activated test using ellagic acid (INTEM), without alterations in Fibrin-based extrinsically activated test (FIBTEM). Two units of fresh frozen plasma, 2 units of platelet concentrate, and 1 g of fibrinogen were administered. The patient was also premedicated with ranitidine, omeprazole and metoclopramide for prophylaxis of pulmonary aspiration of gastric contents and was transferred to the operating room.

A balanced general anesthesia was performed with rapid sequence induction, under standard American Society of Anesthesiologists (ASA) monitoring. The orotracheal intubation was performed using a Mcgrath MAC Video Laryngoscope (medtronic); two high-caliber peripheral venous accesses and an arterial catheter were placed. A bolus of 1 g of tranexamic acid was administered, followed by 1 g infusion over 8 hours. The surgery was performed using electrocautery and biological glue, therefore no significant blood losses were estimated (∼ 300 mL). Fetal extraction was achieved ten minutes after the anesthetic induction. The newborn weighed 3,120 kg and presented an appearance, pulse, grimace, activity, and respiration (APGAR) score of 9/10. The placental deconditioning underwent without immediate intercurrences, after which an oxytocin bolus of 10 IU was administered, followed by an infusion of 15 IU in 500 mL of sodium chloride (NaCl) 0.9%. Throughout the procedure, the patient remained hemodynamically stable, with adequate urine output. Compression bandages were applied over the surgical wound and the patient was extubated without complications.

During the immediate postoperative period, a ROTEM was repeated, which revealed no significant alterations (Fig. 2). The patient was transferred to the intensive care unit (ICU) for surveillance. Not having presented with complications in the first 24 hours and with a platelet count of 47 × 10^9^/L, the patient was transferred to the puerperium ward. The newborn remained under close surveillance in the neonatal special care unit, presenting an initial platelet count of 19 × 10^9^/L. During hospitalization, a gradual normalization of platelet counts was observed, with clinical discharge after 3 days.

## Discussion

Immune thrombocytopenic purpura is an autoimmune disease that affects between 1 and 2 out of every 1,000 pregnant women, accounting for 5% of the cases of pregnancy-related thrombocytopenia. It is characterized by antiplatelet IgG antibodies that bind to platelet membrane glycoproteins, mainly GPIIb/IIIa and GPIb/IX, inducing their destruction in the reticuloendothelial system (predominantly in the spleen). It is usually revealed with persistent thrombocytopenia, with or without peripheral megakaryocytes, and normal or increased normal value of megakaryocytes in the aspirate of the bone marrow, without splenomegaly.^3^ ^6^

This condition may be asymptomatic, diagnosed from routine laboratory tests, or may manifest more rarely as severe cases with petechiae, hemorrhages and hematomas.^7^ Treatment is indicated for platelet counts below 30 × 10^9^/L, with steroids and IgG as first line treatment,^8^ ^9^ and alternatively, thrombopoietin receptors agonists,^10^ rituximab,^11^ or, eventually, splenectomy (occasionally performed and, if necessary, in the 2^nd^ trimester of gestation).^12^ Since there is no treatment with guaranteed effectiveness, many studies have shown that the clinical management of pregnancy, delivery, and puerperium in patients with thrombocytopenia needs a close cooperation of experienced hematologists, obstetricians, anesthesiologists and neonatologist. The treatment of this disorder is largely based on the risk of maternal hemorrhage.^6^ ^13^ ^14^ ^15^

The anesthetic approach of a pregnant woman with ITP is challenging. On the one hand, the locoregional technique is absolutely contraindicated in cases of low platelet count and severe coagulopathy. For platelet counts < 75 × 10^9^/L, the neuroaxial techniques are advised.^16^ On the other hand, general anesthesia is associated with an increased risk of airway hemorrhage due to traumatic injury by the laryngoscope during tracheal intubation, of inadvertent intraocular hemorrhage, of intracranial hemorrhage, and of hemorrhage associated with the surgical procedure.^17^ ^18^ ^19^

The greatest risk in pregnant women with ITP is hemorrhage, which is often associated with surgical incisions (such as episiotomy) and lacerations of the birth canal. There is no increased risk of abruptio placenta or placenta previa in relation to the pregnant population in general, as there is no greater risk of postpartum uterine hemorrhage, since the myometrial contractions produce mechanical hemostasis without significant contribution of platelets.^16^ A platelet count > 50 × 10^9^/L is recommended for delivery, and platelet concentrates should be transfuse if platelet count is  < 20 × 10^9^ L. As shown in Fig. 1, only the IgG cycles resulted in an increase in platelet counts, even though the platelet count had increased, thus the multidisciplinary team discussion about the ideal date for the elective caesarean, which had the lowest risks for the pregnant woman, safeguarding the normal fetus development. Thus, the cesarean section was scheduled for the first day after the completion of an IgG cycle. The ROTEM obtained in the preoperative period guided the therapeutic approach of hemostatic alterations, as suggest by other authors.^20^

In the fetus, complications may arise if the maternal antiplatelet IgG, which crosses the placenta, causes fetal ITP. There is no consistent and reliable correlation between the severity of maternal thrombocytopenia or serum concentration of antiplatelet antibodies and platelet count in the fetus. At delivery, between 10 and 20% of the fetuses present platelet counts < 50 × 10^9^/L, and 5% present platelet counts < 20 × 10^9^/L.^21^ ^22^ During the delivery, the main risk to the fetus, although rare, is intracranial hemorrhage, which can result in severe neurological sequelae, or even death.^23^ Following the delivery, daily monitoring of the platelet value in the newborn is recommended, since it can reduce further in the 4 to 5 days postpartum and is usually normalized at the end of the 1^st^ month.

## Conclusion

It is concluded that all pregnant women with severe thrombocytopenia should be closely monitored for the early detection and treatment of possible complications and reduction of risks of maternal and fetal morbidity and mortality. A preanesthetic evaluation is essential, since side effects of the therapy, including corticotherapy, such as obesity, hypertension, and airway edema, should be considered. The complications associated with the anesthetic technique should be weighed: on the one hand, locoregional techniques have a risk of neuraxial hematoma development and, on the other hand, the possibility of a difficult airway for general anesthesia with high hemorrhagic risk and gastric emptying delay with consequent high risk of pulmonary aspiration.
